# Definition of Carotid Artery Free Floating Thrombus: A Systematic Review and Call for Standardisation of Imaging and Nomenclature

**DOI:** 10.1016/j.ejvsvf.2025.10.002

**Published:** 2025-10-16

**Authors:** Oumaima Aouam, Carolijn J.M. de Bresser, Eline S. van Hattum, W. Marnix de Fijter, Hidde Jongsma, Patrick W.H.E. Vriens, Vincent van Weel, Gert J. de Borst

**Affiliations:** a Department of Vascular Surgery, University Medical Centre Utrecht, Utrecht, the Netherlands; b Department of Vascular Surgery, Elisabeth-Tweesteden Hospital, Tilburg, the Netherlands; c Department of Vascular Surgery, Meander Medical Centre, Amersfoort, the Netherlands; d Board of Directors, Reinier de Graaf Gasthuis, Delft, the Netherlands

**Keywords:** Diagnostic imaging, Free floating thrombus, Reporting standard, Systematic review

## Abstract

**Objective:**

Free floating thrombus in the carotid artery (cFFT) is a rare and poorly understood condition with an unclear definition and diagnostic criteria. This systematic review aimed to propose a reporting standard to ensure diagnostic consistency and a universal definition of cFFT.

**Methods:**

PubMed and EMBASE were systematically searched from inception until 01 May 2025, using terms including “free floating thrombus” and “carotid”, along with imaging modalities. Eligibility criteria included studies on patients with cFFT, diagnosed through well defined imaging criteria. Two authors independently screened eligible literature and extracted data. Study quality was assessed with the Methodological Index for Non-Randomized Studies (MINORS) score. The composite endpoint was the radiological description of cFFT per imaging modality.

**Results:**

A systematic search identified 611 publications, from which 20 were included. These studies were predominantly of low quality, with a mean MINORS score of 9 for non-comparative and 16 for comparative studies. These 20 studies identified 17 distinct imaging based descriptions of cFFT. The descriptions were derived from five different imaging modalities, encompassing both static and dynamic imaging. Computed tomography angiography (CTA) was the most reported modality, followed by duplex ultrasound (DUS). On CTA, cFFT is diagnosed as a thrombus with circumferential flow, proximal attachment to the vessel wall, and extending distally in the lumen. On DUS, cFFT is diagnosed by circumferential flow, attachment to the arterial wall, and synchronous movement with the cardiac cycle.

**Conclusion:**

The published literature lacks high quality studies. It is suggested that the diagnosis of cFFT requires static imaging, preferably with CTA. If static imaging is inconclusive, a dedicated dynamic assessment by DUS, performed by an experienced sonographer, is suggested. The proposed definition aims to standardise the diagnostic workflow for cFFT, thereby ensuring consistent interpretation and unified terminology across research and clinical practice, which should also enhance individual patient data meta-analyses on cFFT in the future.

## HIGHLIGHTS

• There is wide variability in the imaging descriptions of free floating thrombus in the carotid artery (cFFT) and a lack of high quality studies, emphasising the need for standardised diagnostic criteria.
• The authors suggest that the diagnosis of cFFT requires static imaging, preferably computed tomography angiography (CTA); if inconclusive, dedicated dynamic assessment by duplex ultrasound performed by an experienced sonographer is suggested.
• On CTA, cFFT should meet at least two of three criteria: circumferential flow, proximal attachment to the arterial wall, and distal luminal extension.
• The proposed definition aims to standardise the diagnostic workflow for cFFT, ensuring consistent interpretation and unified terminology across research and clinical practice.

## INTRODUCTION

Free floating thrombus in the carotid artery (cFFT) is a rare and poorly understood clinical entity. cFFT was first described by Ehrenfeld in 1966 as an elongated thrombus attached to the arterial wall with circumferential blood flow at its distal end and cyclical motion in alignment with the cardiac cycle.^1^ Symptoms of cFFT can vary from asymptomatic presentation to recurrent carotid territory symptoms, including stroke, transient ischaemic attack, or amaurosis fugax.^2^ Given the overlap of these symptoms with other carotid pathologies, such as carotid artery stenosis or vascular dissection, imaging remains essential.^3^^,^^4^ The reported incidence of cFFT varies considerably among studies, with retrospective cerebral angiogram (DSA) studies reporting rates of 0.4–0.7%, whereas duplex ultrasound (DUS) studies indicate a rate of 0.05%.^5^ This low incidence with DUS may be due to its relatively limited diagnostic accuracy and known interobserver variability.^6^^,^^7^ The 2023 European Society for Vascular Surgery (ESVS) guideline defined cFFT as an elongated thrombus attached to the arterial wall with circumferential blood flow distally.^8^ This definition dates back to 1966; since then, advances in imaging and small studies on cFFT have expanded its understanding.^1^ Therefore, it is pertinent to question whether this characterisation accurately reflects the contemporary understanding of cFFT. This is particularly relevant in a multidisciplinary context, where patients may present to different specialists, typically neurologists and or vascular surgeons. This variation in initial exposure also results in a variety of imaging modalities applied, yet a standardised reporting framework for radiologists is lacking. Furthermore, the 2023 ESVS guidelines exclusively focus on therapeutic recommendations, without specifying preferred imaging modalities or diagnostic criteria.^8^ The 2022 guideline from the Society for Vascular Surgery and the 2021 guidelines from the American Heart Association and the European Stroke Organisation do not address cFFT at all, underscoring the lack of awareness and consensus in the field.^9^, ^10^, ^11^

Accurate diagnosis of cFFT is critical, as therapeutic strategies and prognosis strongly depend on distinction from other carotid pathologies. Current guidelines recommend initial anticoagulation for symptomatic cFFT, whereas symptomatic carotid artery stenosis is typically managed with antiplatelet therapy and surgical intervention.^8^ Given the high risk of recurrent ischaemic events and potential progression to complete occlusion in cFFT, timely and precise differentiation is considered essential to guide evidence based treatment.^12^ Diagnostic precision is also essential for the future development of a cFFT grading scale, which should express the natural course risk of embolisation and stroke, but should also include step up guidance for type of intervention to lower this risk.

Due to the absence of a universally accepted definition of cFFT, and the lack of consensus on the gold standard imaging and radiological reporting, this systematic review aimed to provide a comprehensive overview of existing descriptions and imaging modalities. Based on this overview, the authors sought to propose imaging, work up, and nomenclature standardisation.

## METHODS

This systematic review was conducted according to a predefined protocol that has been prospectively registered in the International Prospective Registry for Systematic Reviews (PROSPERO: CRD 42024593513). The updated 2020 Preferred Reporting Items for Systematic Reviews and Meta-Analysis (PRISMA) checklist was followed (Supplementary Table S1).^13^

PubMed and EMBASE were searched systematically from inception until 01 May 2025 for observational studies and randomised controlled trials meeting the predefined eligibility criteria. The keywords “free floating thrombus” and “carotid”, along with imaging modalities were used (Supplementary Table S2).

### Eligibility criteria, screening process, and data extraction

The full text or abstracts of all potentially relevant studies were retrieved and assessed based on the following eligibility criteria: 1) original data reported in peer reviewed journals in English; 2) studies involving patients diagnosed with cFFT, defined in the methodology or results; 3) diagnosis based on imaging modalities such as ultrasound (US), DUS, computed tomography (CT), computed tomography angiography (CTA), magnetic resonance imaging (MRI), magnetic resonance angiography (MRA), or DSA; and 4) patients aged >18 years. Articles were excluded when: 1) there was an absence of a description of cFFT; 2) the pathology was referred to as intraluminal thrombus (ILT) instead of cFFT; 3) case series had fewer than five patients; and 4) they were cadaveric or animal studies. Two authors (O.A. and C.B.) screened titles and abstracts for eligible studies independently. In case of disagreement, discrepancies were resolved by consensus meetings between both of these authors. The same two authors independently extracted the following study characteristics from the included studies: 1) methods: study design and study year; 2) reported diagnostic imaging modality; and 3) the explicit definitions of cFFT.

### Definitions

Proximal is defined as the part of the cFFT that is situated closer to the trunk of the body, whereas distal refers to the part situated farther away from the trunk.

### Outcome measures

The primary outcome was the description of cFFT per imaging modality irrespective of the modality or the number of modalities used.

### Risk of bias

The methodology of the studies and risk of bias were systematically assessed using the Methodological Index for Non-Randomized Studies (MINORS) score, with a maximum score of 16 for non-comparative and 24 for comparative studies.^14^ A score ≤8 was considered poor quality, 9–14 moderate quality, and 15–16 good quality for non-comparative studies. Cut off points were ≤14, 15–22, and 23–24, respectively, for comparative studies. Authorship of the studies was unblinded during review.

## RESULTS

**Figure 1:**
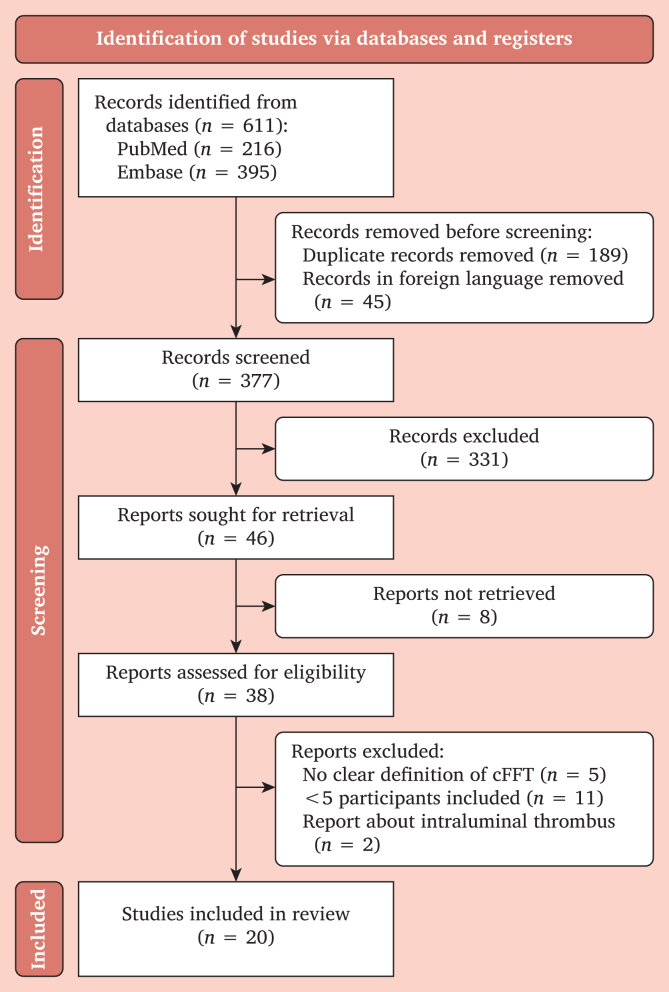
PRISMA flow diagram presenting the number of studies included and excluded at each stage of the review.^13^ cFFT = carotid free floating thrombus.

A total of 611 articles were identified, of which 377 were selected for title and abstract screening. Eight articles were excluded due to the lack of full text availability. Thirty-eight articles underwent full text screening, resulting in the inclusion of 20 articles (Fig. 1, Supplementary Tables S3 and S4).

Table 1 provides an overview of the definitions and imaging modalities used for cFFT diagnosis in the included studies. The 20 included studies, encompassing a total of 581 patients, identified 17 distinct imaging based descriptions of cFFT, with two descriptions being reported in two different studies each. Five distinct imaging modalities were used for initial imaging, with some employing multiple imaging techniques, resulting in a total of 34 reported instances of imaging use. CTA was most frequently used (*n* = 17 of 34, 50%), followed by DUS (*n* = 7 of 34, 21%) and MRA (*n* = 4 of 34, 11%). DSA was used in three study reports (9%). US, CT, and MRI were each equally used (*n* = 1 of 34, 3%).

**Table 1:** Summary of the used descriptions and imaging modalities in diagnosing cFFT.

Author	*n*	Study design	Description of cFFT	Imaging modality
Aboul Nour^25^	66	Single centre retrospective	Loosely associated with the arterial wall and manifesting as a filling defect fully surrounded by flow.	CTA, DUS
Bhogal^30^	7	Single centre retrospective	An elongated thrombus attached to the arterial wall with circumferential blood flow around its most distal aspect, with cyclical motion relating to the cardiac cycle.	CTA, MRI
Chua^33^	25	Single centre retrospective	An elongated thrombus attached to the arterial wall with circumferential blood flow at its distal most aspect, with cyclical motion relating to cardiac cycles.	US
Combe^31^	6	Single centre retrospective	Filling defect on biplane arteriograms.	DSA
Cordier^16^	7	Single centre retrospective	Thrombus with circumferential blood flow of at least five mm length on cervical and intracerebral arteries.	CTA
Dowlatshahi^17^	83	Multicentre retrospective	Either the presence of an intraluminal filling defect that resolves or decreases in size on serial CTA, or the observation of intra-operative pathology during carotid endarterectomy.	CTA
Ferrero^23^	16	Single centre retrospective	Thrombus that originates or is anchored within the artery, exhibiting partial occlusion or an elongated or protrusive morphology. It is characterised by circumferential blood flow around the distal portion and cyclical motion synchronised with the cardiac cycles.	CTA, DSA, DUS, MRA
Gülcü^19^	23	Single centre retrospective	Intra-arterial filling defect totally surrounded by luminal contrast medium in at least two sequential slices and adherent to the wall proximally.	CTA
El Harake^18^	50	Single centre retrospective	A filling defect on CT in two axial planes: the “donut sign” and the “finger sign” and confirmed by a multidisciplinary medical expert.	CTA
Jaberi^29^	25	Single centre prospective	A circular intraluminal filling defect in the axial plane, surrounded by contrast or flow, which resolves or decreases in size on follow up.	CTA, MRA
Lane^27^	6	Single centre prospective	A tongue of thrombus adherent to a non-stenosing atheromatous plaque in the artery, identified by imaging and confirmed through intra-operative findings and histology, with potential complications such as fissures, haemorrhage, or dissection.	CTA, DUS
Müller^24^	62	Single centre retrospective	A contrast filling structure adhering to the carotid wall and extending into the lumen.	CTA, DSA, MRA
Naeem Khan^7^	12	Single centre prospective	An elongated thrombus attached to the arterial wall by a circumferential blood flow. On DUS, an absence of Doppler signals is observed in the region of the thrombus.	CTA, DUS
Onalan^15^	52	Single centre retrospective	A hypodense filling defect within the arterial lumen surrounded by contrast agent on a minimum of three or more consequent source images in the axial images, which were confirmed by sagittal and coronal images.	CT, CTA
Panda^20^	18	Single centre ambispective	A filling defect within the vessel lumen completely surrounded by contrast on at least two contiguous axial source images, producing the classical “donut sign”.	CTA
Pensato^26^	6	Single centre retrospective	A mobile thrombus attached to the carotid wall with a complete circumferential blood flow at its end.	CTA, DUS
Thornhill^21^	23	Single centre retrospective	Endoluminal rounded filling defect in the distal artery on >5 axial images, interpreted as suspicious for intraluminal thrombus in the final report. And resolves of decreased in size on the follow up CTA.	CTA
Tolaymat^28^	6	Single centre retrospective	A mobile thrombus presenting as a linear intraluminal filling defect with a distal string sign distally.	CTA, DUS, MRA
Torres^22^	83	Multicentre prospective	Resolution or a decrease in length (>0.5 mm) of the filling defect on any follow up.	CTA
Vassileva^32^	5	Single centre retrospective	A thrombus originating from or anchored to the artery, causing partial occlusion and or exhibiting an elongated morphology, is characterised by circumferential flow around its distal portion. It presents a relatively homogeneous structure (without hyperechoic zones), a smooth surface, no significant increase in blood velocity, and motion synchronised with the cardiac cycles.	DUS

cFFT = carotid free floating thrombus; CT = computed tomography; CTA = computed tomography angiography; DUS = duplex ultrasound; DSA = digital subtraction angiography; IVT = intravenous thrombectomy; MRA = magnetic resonance angiography; MRI = magnetic resonance imaging; *n* = number of patients diagnosed with cFFT included; US = ultrasound.

Among the included studies, one directly compared the diagnostic accuracy of an imaging modality for cFFT, finding that DUS demonstrated an accuracy of 53% compared with CTA, which served as the reference standard.^7^ The remaining studies did not conduct formal comparative analyses. Although several studies reported a preference for certain modalities or employed defined combinations (e.g., CTA and DUS), the rationale behind these choices was not elaborated upon in the articles.

The most frequently used term across the studies was “circumferential (blood) flow”, or closely related terminology (*n* = 13, 65%). A few articles used specific imaging cut offs, such as a “filling defect on biplane imaging” (*n* = 2, 10%), “filling defect on two axial images” (n = *2*, 10%), “filling defect on ≥ three axial images” (*n* = 1, 5%), and “filling defect on > five axial images” (*n* = 1, 5%). One study (5%) also used the threshold of “circumferential blood flow of at least 5 mm".

The terminology used across the three primary imaging modalities, DUS, CTA, and MRA, show overlap, yet each highlights distinct features (Table 2). CTA most often described cFFT as “circumferential (blood) flow” or “surrounded by contrast” (*n* = 11, 65%) and “attached to the arterial wall” (*n* = 8, 47%). The “doughnut sign” (*n* = 2, 12%) and “finger sign” (*n* = 1, 6%) were also mentioned. On DUS, cFFT was most described as “attached to the arterial wall” (*n* = 6, 86%). Followed by “circumferential (blood) flow” (*n* = 5, 71%) and “motion synchronised with the cardiac cycle” (*n* = 4, 57%). For MRA, common terms included “string”, “elongated” or “extending” (*n* = 3, 75%), and “intraluminal” (*n* = 3, 75%).

**Table 2:** Summary of the frequency of terms used per imaging modality in the diagnosis of cFFT (*n* = 20).

Criteria	No. of articles (*n* = 20)
*CTA*	
Circumferential (blood) flow or surrounded by contrast	11
Attached to the arterial wall	8
Intraluminal	8
Elongated	4
Doughnut sign	2
Finger sign	1
*DUS*	
Attached to the arterial wall	6
Circumferential (blood) flow or surrounded by contrast	5
Motion synchronised with the cardiac cycles or mobile thrombus	4
Elongated	3
Low echogenicity or anechoic appearance	2
Intraluminal	1
*MRA*	
Elongated, string, extension	3
Intraluminal	3
Attached to the arterial wall	2
Circumferential (blood) flow or surrounded by contrast	2

cFFT = carotid free floating thrombus; CTA = computed tomography angiography; DUS = duplex ultrasound; MRA = magnetic resonance angiography.

### Risk of bias

Most studies were single centre (*n* = 18, 90%), while two were multicentre (10%). Most studies employed a retrospective design (*n* = 14, 70%), with fewer using prospective (*n* = 5, 25%) or ambispective (*n* = 1, 5%) approaches. Furthermore, non-comparative studies were more common than comparative (*n* = 15 of 20, 75% *vs*. *n* = 5 of 20, 25%). Notably, no randomised controlled trials were identified. The overall methodological quality was relatively low, with none of the studies being high quality. The mean MINORS scores were 9 for non-comparative and 16 for comparative studies (Supplementary Table S5).

## DISCUSSION

This review aimed to provide an overview of the existing literature regarding the most frequently used imaging modalities and their description of cFFT. The authors propose that the diagnosis of cFFT should include static imaging, preferably by CTA, with a 0.9 mm slice thickness, assessed in three planes. It should demonstrate at least two of the following three key findings: 1) the presence of a thrombus with circumferential flow on the axial plane; 2) the thrombus should be attached proximally to the vessel wall on the sagittal plane; and or 3) the thrombus should extend distally within the arterial lumen in the sagittal or coronal plane. If fewer than two criteria are met on static imaging and cFFT cannot be reliably excluded, a dedicated dynamic assessment, preferably with DUS performed by an experienced sonographer, is recommended. On DUS, it should include at least two of the following three key findings: 1) a thrombus with circumferential flow; 2) attachment to the arterial wall; and or 3) synchronous movement with the cardiac cycle. With this proposed definition and diagnostic approach, a homogeneous group of cFFT patients can be identified, enabling follow up strategies, prognosis, and treatment recommendations, providing a foundation for future perspectives in retrospective or prospective studies.

The study demonstrated that across different imaging modalities, a range of descriptions were used for cFFT, some of which overlapped with features characteristic of atherosclerotic plaques or carotid artery stenosis, potentially leading to diagnostic ambiguity. For instance, the term “string sign”, which is used in both static and dynamic imaging to describe cFFT, causing a distal filling defect that is not attached to the vessel wall, is also commonly applied in the literature to denote residual contrast flow through a high grade stenosed carotid artery.^34^ Therefore, the term “string sign” should be avoided in the context of cFFT due to its dual meanings.

**Figure 2:**
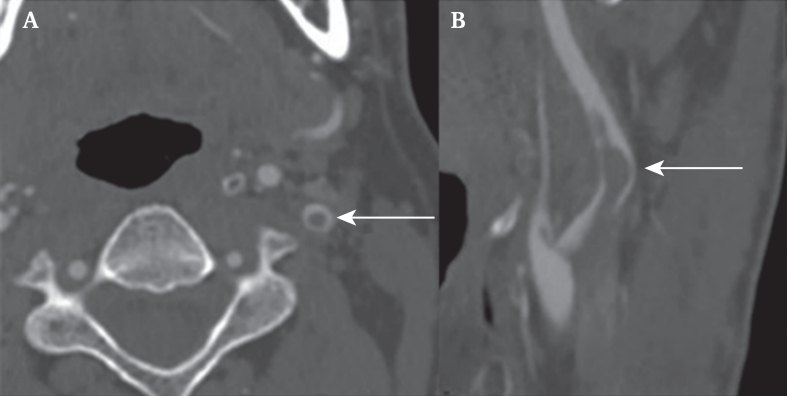
Computed tomography angiography demonstrating a free floating carotid thrombus in the left internal carotid artery with (A) the “doughnut sign” on the axial plane, and (B) the “finger sign” on the coronal plane, both indicated by the arrow.^24^

The “doughnut sign” and “finger sign” are other metaphors exclusively used in static imaging to describe cFFT, possibly causing misinterpretation. The “doughnut sign” refers to an intraluminal filling defect with circumferential flow (Fig. 2a). The “finger sign” describes a thrombus causing a distal filling defect unattached to the vascular wall, resembling an intraluminal string or finger (Fig. 2b).^18^^,^^28^ Metaphorical terms can enhance intercollegiate communication by providing intuitive visual impressions. However, in clinical guidelines, scientific reporting, and education, it is crucial to use terminology that is clearly defined and reproducible. Therefore, the use of metaphorical terms must be accompanied by clear definitions to minimise the risk of misinterpretation.

In the literature, the diagnosis of cFFT is primarily radiological, with CTA being the most used modality, characterised by key features such as circumferential flow, attachment to the arterial wall, and distal extension into the lumen. However, there are cases where one of these features is absent on CTA, yet cFFT has still been confirmed pathologically.^27^ When key features are absent on CTA, cFFT may mimic ILT, carotid atherosclerosis, or carotid artery dissection. Although ILT is often interchangeably used with cFFT, it encompasses various intraluminal thrombus types, such as mural thrombi, making ILT a broader term that includes cFFT but is not limited to it.^35^, ^36^, ^37^, ^38^ cFFT mobility can create a false appearance of full attachment to the vessel wall on CTA, resembling a soft plaque, mural thrombus, or a (lodged) embolus, which typically presents as an abrupt vessel cut off, a meniscus sign, or an isolated intraluminal filling defect, occasionally with layered or tapered flow patterns.^39^ Distinction between plaque and thrombus can be made as cFFT appears as a smooth, elongated structure extending into the lumen, whereas (ulcerated) plaques typically exhibit a crater like, irregular surface that projects perpendicularly or obliquely into the lumen and remains localised, without significant distal extension.^3^^,^^40^ Furthermore, distinction between plaque and thrombus can be made over time, as a persisting static filling defect on follow up scans after antithrombotic therapy is typically indicative of plaque.^21^ Finally, carotid artery dissection should also be considered in the differential diagnosis. Typical features such as a tapered stenosis, hypoechoic wall thickening from intramural haematoma, an intimal flap with double lumen, or a pseudoaneurysm with “yin-yang” flow are characteristic of dissection and not typically seen in cFFT.^41^ Moreover, dissections lack circumferential contrast flow and concentric narrowing on CTA, further aiding distinction from cFFT.

DSA is the gold standard for evaluating carotid artery stenosis. However, its invasive nature and procedural complexity have led to an increased reliance on non-invasive modalities such as DUS and CTA now serving for first assessment.^42^ This review reflects this changing imaging paradigm, demonstrating that CTA and dedicated DUS are now the predominant techniques for detecting cFFT in clinical practice. CTA appears to be particularly well suited and may reasonably be regarded as the current reference standard for diagnosis. As previously noted, cFFT may mimic other carotid pathologies, mainly due to its mobility; therefore, the incorporation of an additional dedicated dynamic imaging modality may be warranted when initial static imaging findings are inconclusive. This recommendation is based on expert opinion and evidence from imaging studies in carotid artery disease in general, as specific studies in cFFT are lacking. DUS allows for precise assessment of stenosis severity, morphological characteristics, and the measurement of thrombus motion synchronised with cardiac rhythm, thereby aiding in the differentiation from other carotid pathologies.^32^^,^^43^ However, basic DUS screening has demonstrated limited diagnostic accuracy, with reported rates as low as 53% in patients with cFFT.^7^ Although no studies have specifically evaluated the accuracy of DSA for cFFT, existing evidence indicates that DSA is also unreliable in the assessment of carotid artery stenosis.^44^ Therefore, basic dynamic imaging should not be relied upon as a standalone modality for diagnosing or excluding cFFT, due to its relatively low diagnostic accuracy. The authors propose that once the predefined static imaging criteria for cFFT are met, additional dynamic imaging is unnecessary. Static modalities demonstrate superior reproducibility and markedly lower interobserver variability, thereby ensuring greater consistency and reliability during follow up assessments.^6^ Moreover, static imaging is highly standardisable and amenable to artificial intelligence driven quantitative scoring.^45^ As CTA is minimally invasive and widely accessible due to its cost effectiveness, this imaging modality is first choice for static imaging. Alternatively, MRA can be considered for static imaging in younger patients, to avoid radiation exposure, in patients with contrast agent allergy or calcium deposits within the thrombus.^5^^,^^46^ CTA studies of coronary artery stenosis have shown that calcium deposits can create blooming artifacts and beam hardening, leading to an overestimation of stenosis severity, thereby hindering cFFT diagnosis.^5^^,^^46^^,^^47^ Furthermore, MRA uses gadolinium based contrast agents, which are generally safer due to their lack of nephrotoxicity, unlike the iodine based agents used in CTA.^46^^,^^48^

Several emerging imaging modalities have been developed over the last few decades, including intravascular ultrasound (IVUS) and optical coherence tomography (OCT), which allow detailed visualisation of the vessel wall and intraluminal pathology, enabling differentiation between thrombotic material and plaque component.^49^ OCT, in particular, provides high resolution visualisation of thrombus and enables characterisation of cFFT composition, which may range from mainly thrombotic to debris rich lesions with cholesterol crystals.^49^ Such heterogeneity may explain the variable response to anticoagulation therapy that is observed clinically.^49^ However, the clinical added value, safety, and cost effectiveness of IVUS and OCT in the diagnosis and management of cFFT remain to be established. More specifically, these intra-arterial techniques have long been neglected due to the fear of dislodging a thrombus when passing the cFFT, resulting in distal thrombo-embolisation and subsequent massive stroke. Future research using these modalities may provide critical insights into the pathophysiology, aid in the diagnosis, and inform optimal treatment strategies for these complex lesions.

Lastly, in studies using CTA in the diagnostic work up of cFFT, a threshold is often applied, requiring a minimum number of axial slices or a specific thrombus size in millimetres, for diagnosis. These criteria are supported by limited scientific evidence and vary considerably between studies, leading to diagnostic inconsistency; the same thrombus may be classified as cFFT or as mural thrombus.^15^^,^^16^^,^^19^^,^^21^^,^^22^ Given that cFFT can be reliably diagnosed when the thrombus is completely surrounded by contrast, particularly when combined with DUS, the need for such numerical thresholds is questionable. Considering current evidence, the authors advocate the use of descriptive imaging criteria alone, which more accurately reflect the present state of knowledge.

This literature research was comprehensive and used predefined selection criteria for study inclusion. The search strategy was unrestricted by publication date, data extraction was conducted independently by two reviewers, and a thorough critical appraisal of the included studies was conducted. The current literature has several limitations. Firstly, the limited quality of included studies, with a mean MINORS score of 9 for non-comparative studies and 15 for comparative studies, indicating relatively poor study quality. Secondly, only case series with five or more patients were included, excluding case reports, thereby limiting the risk of publication bias. Thirdly, studies using the term ILT were excluded, as this is an umbrella term for both cFFT and thrombi fully attached to the vascular wall, therefore possibly excluding some cFFT cases.

### Conclusion

The published literature lacks high quality studies. It is suggested that the diagnosis of cFFT requires static imaging, preferably with CTA, on which cFFT is shown with circumferential flow, proximal attachment to the arterial wall, and distal luminal extension. If static imaging is inconclusive, dedicated dynamic assessment by DUS, performed by an experienced sonographer, is suggested. This definition facilitates a more accurate diagnosis of cFFT by distinguishing it from other carotid pathologies. Furthermore, it contributes to the establishment of a standardised diagnostic workflow for cFFT, thereby ensuring consistent interpretation and unified terminology across research and clinical practice, which should also enhance individual patient data meta-analyses on cFFT in the future.

## FUNDING

This research received no specific grant from any funding agency in the public, commercial, or not for profit sectors.

## CONFLICT OF INTEREST

The authors declare that they have no conflict of interest.
