# Supplementary material for: Definition of Carotid Artery Free Floating Thrombus: A Systematic Review and Call for Standardisation of Imaging and Nomenclature
Source: EJVES Vasc Forum. 2025 Oct 16;64:199–207. doi: 10.1016/j.ejvsvf.2025.10.002 (PMC12670957; doi:10.1016/j.ejvsvf.2025.10.002)
Supplement: Multimedia component 1 [file mmc1.pdf]

Definition of the carotid free-floating thrombus: a systematic review and call for standardization in nomenclature.

Supplemental Material

Supplementary table 1. PRISMA 2020 Checklist

Section and Topic	Item #	Checklist item	Location where item is reported
**TITLE**			
**Title**	1	Identify the report as a systematic review.	Page 1
**ABSTRACT**			
**Abstract**	2	See the PRISMA 2020 for Abstracts checklist.	Page 2
**INTRODUCTION**			
**Rationale**	3	Describe the rationale for the review in the context of existing knowledge.	Page 2 – 3
**Objectives**	4	Provide an explicit statement of the objective(s) or question(s) the review addresses.	Page 3 – 4
**METHODS**			
**Eligibility criteria**	5	Specify the inclusion and exclusion criteria for the review and how studies were grouped for the syntheses.	Page 4
**Information sources**	6	Specify all databases, registers, websites, organizations, reference lists and other sources searched or consulted to identify studies. Specify the date when each source was last searched or consulted.	Page 4
**Search strategy**	7	Present the full search strategies for all databases, registers and websites, including any filters and limits used.	Page 5 of Supplementary table 2
**Selection process**	8	Specify the methods used to decide whether a study met the inclusion criteria of the review, including how many reviewers screened each record and each report retrieved, whether they worked independently, and if applicable, details of automation tools used in the process.	Page 5
**Data collection process**	9	Specify the methods used to collect data from reports, including how many reviewers collected data from each report, whether they worked independently, any processes for obtaining or confirming data from study investigators, and if applicable, details of automation tools used in the process.	Page 5
**Data items**	10a	List and define all outcomes for which data were sought. Specify whether all results that were compatible with each outcome domain in each study were sought (e.g. for all measures, time points, analyses), and if not, the methods used to decide which results to collect.	Page 5
	10b	List and define all other variables for which data were sought (e.g. participant and intervention characteristics, funding sources). Describe any assumptions made about any missing or unclear information.	Page 5
**Study risk of bias assessment**	11	Specify the methods used to assess risk of bias in the included studies, including details of the tool(s) used, how many reviewers assessed each study and whether they worked independently, and if applicable, details of automation tools used in the process.	Page 5

**Effect measures**	12	Specify for each outcome the effect measure(s) (e.g. risk ratio, mean difference) used in the synthesis or presentation of results.	NA
**Synthesis methods**	13a	Describe the processes used to decide which studies were eligible for each synthesis (e.g. tabulating the study intervention<br>characteristics and comparing against the planned groups for each synthesis (item #5)).	NA
	13b	Describe any methods required to prepare the data for presentation or synthesis, such as handling of missing summary statistics, or data conversions.	NA
	13c	Describe any methods used to tabulate or visually display results of individual studies and syntheses.	NA
	13d	Describe any methods used to synthesise results and provide a rationale for the choice(s). If meta-analysis was performed, describe the model(s), method(s) to identify the presence and extent of statistical heterogeneity, and software package(s) used.	NA
	13e	Describe any methods used to explore possible causes of heterogeneity among study results (e.g. subgroup analysis, meta-regression).	NA
	13f	Describe any sensitivity analyses conducted to assess robustness of the synthesised results.	NA
**Reporting bias assessment**	14	Describe any methods used to assess risk of bias due to missing results in a synthesis (arising from reporting biases).	Page 6
**Certainty assessment**	15	Describe any methods used to assess certainty (or confidence) in the body of evidence for an outcome.	NA
**RESULTS**			
**Study selection**	16a	Describe the results of the search and selection process, from the number of records identified in the search to the number of studies included in the review, ideally using a flow diagram (see fig 1).	Figure 1 and Supplementary table 5.
	16b	Cite studies that might appear to meet the inclusion criteria, but which were excluded, and explain why they were excluded.	NA
	17	Cite each included study and present its characteristics.	Supplementary table 4, page 7.
	18	Present assessments of risk of bias for each included study.	Supplementary, table 5, page 8.
	19	For all outcomes, present, for each study: (a) summary statistics for each group (where appropriate) and (b) an effect estimate and its precision (e.g. confidence/credible interval), ideally using structured tables or plots.	NA
	20a	For each synthesis, briefly summarize the characteristics and risk of bias among contributing studies.	Page 10 and supplementary table 5, page 8.

	20b	Present results of all statistical syntheses conducted. If meta-analysis was done, present for each the summary estimate and its precision (e.g. confidence/credible interval) and measures of statistical heterogeneity. If comparing groups, describe the direction of the effect.	NA
	20c	Present results of all investigations of possible causes of heterogeneity among study results.	NA
	20d	Present results of all sensitivity analyses conducted to assess the robustness of the synthesised results.	NA
	21	Present assessments of risk of bias due to missing results (arising from reporting biases) for each synthesis assessed.	Page 10 and supplementary table 5, page 8.
	22	Present assessments of certainty (or confidence) in the body of evidence for each outcome assessed.	NA
**DISCUSSION**			
	23a	Provide a general interpretation of the results in the context of other evidence.	Page 11
	23b	Discuss any limitations of the evidence included in the review.	Page 11 – 14
	23c	Discuss any limitations of the review processes used.	Page 14
	23d	Discuss implications of the results for practice, policy, and future research.	Page 13 - 14
**OTHER INFORMATION**			
**Registration and protocol**	24a	Provide registration information for the review, including register name and registration number, or state that the review was not registered	Page 5
	24b	Indicate where the review protocol can be accessed, or state that a protocol was not prepared	NA
	24c	Describe and explain any amendments to information provided at registration or in the protocol.	NA
	25	Describe sources of financial or non-financial support for the review, and the role of the funders or sponsors in the review.	Page 15
	26	Declare any competing interests of review authors.	Page 15
**Availability of data, code, and other materials**	27	Report which of the following are publicly available and where they can be found: template data collection forms; data extracted from included studies; data used for all analyses; analytic code; any other materials used in the review.	NA

Supplementary table 2. Search strategy

MEDLINE via PubMed (01/05/2025: 216 results)	
1.	("free-floating thromb*"[Title/Abstract] OR "floating thrombus" [Title/Abstract] OR "free floating thromb*"[Title/Abstract])
2.	("Computed Tomography Angiography"[MeSH Terms] OR "Arteriography"[title/abstract] OR "CTA" [Title/Abstract] OR "Angiography" [Title/Abstract] OR "Duplex"[Title/Abstract] OR "Ultrasound"[Title/Abstract] OR "Magnetic Resonance"[Title/Abstract] OR "radiological" [Title/Abstract] OR "imaging" [Title/Abstract])
3.	#1 AND #2
EMBASE (01/05/2025: 395 results)	
1.	('free-floating thrombus':ti,ab OR 'floating thrombus':ti,ab OR 'free floating thromb*':ti,ab)
2.	('computed tomography angiography':ti,ab OR 'arteriography':ti,ab OR 'cta':ti,ab OR 'angiography':ti,ab OR 'duplex':ti,ab OR 'ultrasound':ti,ab OR 'magnetic resonance':ti,ab OR 'radiological':ti,ab OR 'imaging':ti,ab)
3.	#1 AND #2

Supplementary table 3. Full-text evaluation

Reason for exclusion of studies (n = 18)
1. ≤ 5 participants included in studie (n = 11)
2. No clear definition of carotid free-floating thrombus provided (n = 5)
3. Report about intraluminal thrombus (n = 2)
**Included studies (N = 20*)**
*See Supplementary table 4 for a full overview of included articles.

Supplementary table 4. Overview of the characteristics of the included articles.

Author	Country	Publication year	Start year study	End year study	Study design	Comparative/ Non-comparative	MINORS score*
Aboul Nour[1]	USA	2024	2015	2023	Single-center retrospective study	Comparative	12 (24)
Bhogal[2]	GER	2020	2008	2019	Single-center retrospective study	Non-comparative	9 (16)
Chua[3]	SG	2012	1999	2009	Single-center retrospective study	Non-comparative	8 (16)
Combe[4]	FR	1990	1981	1988	Single-center retrospective study	Non-comparative	9 (16)
Cordier[5]	FR	2012	2001	2011	Single-center retrospective study	Non-comparative	9 (16)
Dowlatshahi[6]	CAN	2022	-	-	Multicenter prospective observational study	Non-comparative	11 (16)
El Harake[7]	FR	2023	2017	2019	Single-center retrospective study	Comparative	14 (24)
Ferrero[8]	IT	2011	2000	2008	Single-center retrospective study	Non-comparative	9 (16)
Gülcü[19]	TR	2014	2012	2014	Single-center retrospective study	Non-comparative	7 (16)
Jaberi[9]	GER	2013	2008	2013	Single-center prospective study	Comparative	19 (24)
Lane[10]	UK	2010	2007	2009	Single-center prospective study	Non-comparative	4 (16)
Müller[11]	GER	2022	2005	2020	Single-center retrospective study	Non-comparative	9 (16)
Naeem Khan[12]	PK	2022	2022	2022	Single-center prospective study	Non-comparative	13 (16)
Onalan[13]	TR	2024	2020	2022	Single-center retrospective study	Non-comparative	11 (16)
Panda[14]	IN	2022	2020	2020	Single-center ambispective study	Non-comparative	10 (16)
Pensato[15]	IT	2023	2020	2021	Single-center retrospective study	Non-comparative	11 (16)
Thornhill[16]	CAN	2014	2008	2012	Single-center retrospective study	Comparative	14 (24)
Tolaymat[17]	USA	2019	2016	2018	Single-center retrospective study	Non-comparative	6 (16)
Torres[18]	CAN	2021	2015	2019	Multicenter prospective observational study	Comparative	20 (24)
Vassileva[20]	BG	2014	2009	2013	Single-center retrospective study	Non-comparative	10 (16)

BG: Bulgaria; CAN: Canada; FR: France; GER: Germany; IN: India; IT: Italy; MINORS: Methodological Index for Non-Randomized Studies; PK: Pakistan; SG: Singapore; TR: Turkey; UK: United Kingdom; USA: United States of America.
* The maximum score is 16 for non-comparative studies and 24 for comparative studies. See Supplementary table 5 for a full assessment of each article.

Supplementary table 5. The methodological index for non-randomized studies (MINORS) quality scores of the included studies.

	Aboul Nour [1]	Bhogal [2]	Chua [3]	Combe [4]	Cordier [5]	Dowlatshahi [6]	El Harake [7]	Ferrero [8]	Gülcü [9]	Jaberi [10]	Lane [11]	Muller [12]	Naeem Khan [13]	Onalan [14]	Panda [15]	Pensato [16]	Thornhill [17]	Tolaymat [18]	Torres [19]	Vassileva [20]
(1) The study precisely addressed a specific question in line with existing	2	1	2	2	2	2	2	2	1	2	0	2	2	2	2	2	2	2	2	2
(2) Included all eligible patients without exclusions during the study period.	2	2	2	2	2	2	1	1	2	2	1	1	2	1	2	1	1	0	1	1
(3) Collected data prospectively based on a predefined protocol.	0	1	0	1	0	2	1	1	2	2	1	1	2	1	1	1	1	1	2	1
(4) Established clear criteria for evaluating main outcomes and endpoints.	2	2	0	0	2	2	2	2	0	2	0	2	1	2	1	2	2	1	2	2
(5) Ensured unbiased assessments through blinded evaluations.	0	0	0	0	0	0	0	0	0	1	0	0	0	2	0	1	2	0	2	1
(6) Ensured the follow-up duration aligns with the study's aims.	2	2	2	2	2	2	2	2	1	2	1	2	2	2	2	1	2	1	2	2
(7) Maintained a follow-up loss of less than 5% or included all patients.	0	1	2	2	1	1	0	1	1	2	1	1	2	1	2	1	1	1	1	1
(8) Calculated study size considering statistical relevance.	0	0	0	0	0	0	0	0	0	0	0	0	2	0	0	2	1	0	2	0
**For comparative studies also the next points:**																				
(9) Ensured an adequate control group in comparative studies.	1						2			2							0		0	
(10) Managed control and study groups simultaneously.	1						1			1							0		2	
(11) Ensured baseline equivalence between groups.	0						1			1							0		2	
(12) Utilized appropriate statistical analysis methods for reliability.	2						2			2							2		2	
**TOTAL MINORS score**	12	9	8	9	9	11	14	9	7	19	4	9	13	11	10	11	14	6	20	10
**Maximum possible score**	24	16	16	16	16	16	24	16	16	24	16	16	16	16	16	16	24	16	24	16

MINORS: Methodological index for non-randomized studies.

The items are scored 0 (not reported), 1 (reported but inadequate) or 2 (reported and adequate). The global ideal score being 16 for non-comparative studies and 24 for comparative studies.

Reference list of included articles

1. Aboul-Nour H, Alshaer Q, Khalid FC, et al. Anticoagulants versus Antiplatelet Treatment in the Medical Management of Carotid Floating Thrombus. *J Stroke Cerebrovasc Dis*. 2024;33(7):107760. doi:10.1016/j.jstrokecerebrovasdis.2024.107760

2. Bhogal P, AlMatter M, Aguilar Pérez M, Bäzner H, Henkes H, Hellstern V. Carotid Stenting as Definitive Treatment for Free Floating Thrombus—Review of 7 Cases. *Clin Neuroradiol*. 2021;31(2):449-455. doi:10.1007/s00062-020-00898-y

3. Chua HC, Lim T, Teo BC, Phua Z, Eng J. Free-floating thrombus of the carotid artery detected on carotid ultrasound in patients with cerebral infarcts: a 10-year study. *Ann Acad Med Singap*. 2012;41(9):420-424.

4. Combe J, Poinsard P, Besancenot J, et al. Free-floating Thrombus of the Extracranial Internal Carotid Artery. *Ann Vasc Surg*. 1990;4(6):558-562. doi:10.1016/S0890-5096(06)60839-X

5. M. M. M Cordier. Floating arterial thrombus in acute stroke treated with intravenous thrombolysis: Seven cases with mixed outcome . *Cerebrovascular Diseases*. 2012;33(Suppl. 2):1-2. doi:10.1159/000339538

6. Dowlatshahi D, Lum C, Menon BK, et al. Aetiology of extracranial carotid free-floating thrombus in a prospective multicentre cohort. *Stroke Vasc Neurol*. 2023;8(3):194-196. doi:10.1136/svn-2022-001639

7. El Harake S, Doche E, Bertolino J, et al. Symptomatic Carotid Free-Floating Thrombus: About Management of 50 Cases in a Referral Neurovascular Center. *J Clin Med*. 2023;12(23):7238. doi:10.3390/jcm12237238

8. Ferrero E, Ferri M, Viazzo A, et al. Free-floating thrombus in the internal carotid artery: Diagnosis and treatment of 16 cases in a single center. *Ann Vasc Surg*. 2011;25(6):805-812. doi:10.1016/j.avsg.2011.02.030

9. Jaberi A, Lum C, Stefanski P, et al. Computed tomography angiography intraluminal filling defect is predictive of internal carotid artery free-floating thrombus. *Neuroradiology*. 2014;56(1):15-23. doi:10.1007/s00234-013-1298-7

10. Lane TRA, Shalhoub J, Perera R, et al. Diagnosis and surgical management of free-floating thrombus within the carotid artery. *Vasc Endovascular Surg*. 2010;44(7):586-593. doi:10.1177/1538574410375312

11. Müller MD, Raptis N, Mordasini P, et al. Natural history of carotid artery free-floating thrombus—A single center, consecutive cohort analysis. *Front Neurol*. 2022;13. doi:10.3389/fneur.2022.993559

12. Naeem Khan MN, Ahmed A, Zafar I, Akhtar S, Aurangzeb MH, Khan A. The Diagnostic Accuracy of Carotid Doppler in Detecting Anechoic Thrombus Against CT Angiography as the Gold Standard. *Cureus*. 2022;14(7):e26951. doi:10.7759/cureus.26951

13. Onalan A, Gurkas E, Kursad Akpinar C, et al. Safety and effectiveness of anticoagulation in the management of acute stroke and transient ischemic attack due to intracranial and extracranial non-occlusive thrombus. *J Clin Neurosci*. 2024;124:47-53. doi:10.1016/j.jocn.2024.04.012

14. Panda S, Tiwari S, Pamnani J, et al. Large Vessel Occlusions By Free Floating Thrombi in Strokes During the COVID-19 pandemic- A Single Center Observational Study. *Neurol India*. 2022;70(2):623-632. doi:10.4103/0028-3886.344655

15. Pensato U, Forlivesi S, Gentile M, et al. Carotid free-floating thrombus in COVID-19: a cerebrovascular disorder of cytokine storm-related immunothrombosis. *Neurol Sci*. 2023;44(6):1855-1860. doi:10.1007/s10072-023-06682-3

16. Thornhill RE, Lum C, Jaberi A, et al. Can Shape Analysis Differentiate Free-floating Internal Carotid Artery Thrombus from Atherosclerotic Plaque in Patients Evaluated with CTA for Stroke or Transient Ischemic Attack? *Acad Radiol*. 2014;21(3):345-354. doi:10.1016/j.acra.2013.11.011

17. Tolaymat B, Irizarry K, Reif M, et al. Considerations beyond Stenosis for Carotid Endarterectomy in Treating Free-Floating Thrombus of the Carotid Artery. *Ann Vasc Surg*. 2019;60:221-228. doi:10.1016/j.avsg.2019.02.024

18. Torres C, Lum C, Puac-Polanco P, et al. Differentiating Carotid Free-Floating Thrombus From Atheromatous Plaque Using Intraluminal Filling Defect Length on CTA. *Neurology*. 2021;97(8):e785-e793. doi:10.1212/WNL.0000000000012368

19. Gülcü A, Gezer NS, Men S, Öz D, Yaka E, Öztürk V. Management of free-floating thrombus within the arcus aorta and supra-aortic arteries. *Clin Neurol Neurosurg*. 2014;125:198-206. doi:10.1016/j.clineuro.2014.08.008

20. Vassileva E, Daskalov M, Stamenova P. Free-Floating Thrombus in Stroke Patients with Nonstenotic Internal Carotid Artery-An Ultrasonographic Study. *J Clin Ultrasound*. 2015;43(1):34-38. doi:10.1002/jcu.22172
