# Supplementary material for: Definition of Carotid Artery Free Floating Thrombus: A Systematic Review and Call for Standardisation of Imaging and Nomenclature
Source: EJVES Vasc Forum. 2025 Oct 16;64:199–207. doi: 10.1016/j.ejvsvf.2025.10.002 (PMC12670957; doi:10.1016/j.ejvsvf.2025.10.002)
Supplement: Multimedia component 3 [file mmc3.pdf]

Supplementary Table S2. Search strategy.

MEDLINE via PubMed (01/05/2025: 216 results)

1.	**("free-floating thromb*"[Title/Abstract] OR "floating thrombus" [Title/Abstract] OR "free floating thromb*"[Title/Abstract])**
2.	**("Computed Tomography Angiography"[MeSH Terms] OR "Arteriography"[title/abstract] OR "CTA" [Title/Abstract] OR "Angiography" [Title/Abstract] OR "Duplex"[Title/Abstract] OR "Ultrasound"[Title/Abstract] OR "Magnetic Resonance"[Title/Abstract] OR "radiological" [Title/Abstract] OR "imaging" [Title/Abstract])**
3.	#1 AND #2

EMBASE (01/05/2025: 395 results)

1.	**('free-floating thrombus':ti,ab OR 'floating thrombus':ti,ab OR 'free floating thromb*':ti,ab)**
2.	**('computed tomography angiography':ti,ab OR 'arteriography':ti,ab OR 'cta':ti,ab OR 'angiography':ti,ab OR 'duplex':ti,ab OR 'ultrasound':ti,ab OR 'magnetic resonance':ti,ab OR 'radiological':ti,ab OR 'imaging':ti,ab)**
3.	#1 AND #2
