# Supplementary material for: Definition of Carotid Artery Free Floating Thrombus: A Systematic Review and Call for Standardisation of Imaging and Nomenclature
Source: EJVES Vasc Forum. 2025 Oct 16;64:199–207. doi: 10.1016/j.ejvsvf.2025.10.002 (PMC12670957; doi:10.1016/j.ejvsvf.2025.10.002)
Supplement: Multimedia component 4 [file mmc4.pdf]

Supplementary Table S3. Full text evaluation.

Reason for exclusion of studies (n = 18)
1. ≤ 5 participants included in studies (n = 11)
2. No clear definition of carotid free-floating thrombus provided (n = 5)
3. Report about intraluminal thrombus (n = 2)
**Included studies (N = 20*)**
**See Supplementary table 4 for a full overview of included articles.*
