# Supplementary material for: Definition of Carotid Artery Free Floating Thrombus: A Systematic Review and Call for Standardisation of Imaging and Nomenclature
Source: EJVES Vasc Forum. 2025 Oct 16;64:199–207. doi: 10.1016/j.ejvsvf.2025.10.002 (PMC12670957; doi:10.1016/j.ejvsvf.2025.10.002)
Supplement: Multimedia component 5 [file mmc5.pdf]

Supplementary Table S4. Overview of the characteristics of the included articles.

Author	Country	Publication year	Start year study	End year study	Study design	Comparative/ Non-comparative	MINORS score*
Aboul Nour[1]	USA	2024	2015	2023	Single-center retrospective study	Comparative	12 (24)
Bhogal[2]	GER	2020	2008	2019	Single-center retrospective study	Non-comparative	9 (16)
Chua[3]	SG	2012	1999	2009	Single-center retrospective study	Non-comparative	8 (16)
Combe[4]	FR	1990	1981	1988	Single-center retrospective study	Non-comparative	9 (16)
Cordier[5]	FR	2012	2001	2011	Single-center retrospective study	Non-comparative	9 (16)
Dowlatshahi[6]	CAN	2022	-	-	Multicenter prospective observational study	Non-comparative	11 (16)
El Harake[7]	FR	2023	2017	2019	Single-center retrospective study	Comparative	14 (24)
Ferrero[8]	IT	2011	2000	2008	Single-center retrospective study	Non-comparative	9 (16)
Gülcü[19]	TR	2014	2012	2014	Single-center retrospective study	Non-comparative	7 (16)
Jaberi[9]	GER	2013	2008	2013	Single-center prospective study	Comparative	19 (24)
Lane[10]	UK	2010	2007	2009	Single-center prospective study	Non-comparative	4 (16)
Müller[11]	GER	2022	2005	2020	Single-center retrospective study	Non-comparative	9 (16)
Naeem Khan[12]	PK	2022	2022	2022	Single-center prospective study	Non-comparative	13 (16)
Onalan[13]	TR	2024	2020	2022	Single-center retrospective study	Non-comparative	11 (16)
Panda[14]	IN	2022	2020	2020	Single-center ambispective study	Non-comparative	10 (16)
Pensato[15]	IT	2023	2020	2021	Single-center retrospective study	Non-comparative	11 (16)
Thornhill[16]	CAN	2014	2008	2012	Single-center retrospective study	Comparative	14 (24)

Tolaymat[17]	USA	2019	2016	2018	Single-center retrospective study	Non-comparative	6 (16)
Torres[18]	CAN	2021	2015	2019	Multicenter prospective observational study	Comparative	20 (24)
Vassileva[20]	BG	2014	2009	2013	Single-center retrospective study	Non-comparative	10 (16)

BG: Bulgaria; CAN: Canada; FR: France; GER: Germany; IN: India; IT: Italy; MINORS: Methodological Index for Non-Randomized Studies; PK: Pakistan; SG: Singapore; TR: Turkey; UK: United Kingdom; USA: United States of America.

** The maximum score is 16 for non-comparative studies and 24 for comparative studies. See Supplementary table 5 for a full assessment of each article.*
