# Supplementary material for: Definition of Carotid Artery Free Floating Thrombus: A Systematic Review and Call for Standardisation of Imaging and Nomenclature
Source: EJVES Vasc Forum. 2025 Oct 16;64:199–207. doi: 10.1016/j.ejvsvf.2025.10.002 (PMC12670957; doi:10.1016/j.ejvsvf.2025.10.002)
Supplement: Multimedia component 6 [file mmc6.pdf]

Supplementary Table S5. The methodological index for non-randomised studies (MINORS) quality scores of the included studies.

	Aboul Nour [1]	Bhogal [2]	Chua [3]	Combe [4]	Cordier [5]	Dowlatshahi [6]	El Harake [7]	Ferrero [8]	Gülcü [9]	Jaberi [10]	Lane [11]	Muller [12]	Naeem Khan [13]	Onalan [14]	Panda [15]	Pensato [16]	Thornhill [17]	Tolaymat [18]	Torres [19]	Vassileva [20]
(1) The study precisely addressed a specific question in line with existing	2	1	2	2	2	2	2	2	1	2	0	2	2	2	2	2	2	2	2	2
(2) Included all eligible patients without exclusions during the study period.	2	2	2	2	2	2	1	1	2	2	1	1	2	1	2	1	1	0	1	1
(3) Collected data prospectively based on a predefined protocol.	0	1	0	1	0	2	1	1	2	2	1	1	2	1	1	1	1	1	2	1
(4) Established clear criteria for evaluating main outcomes and endpoints.	2	2	0	0	2	2	2	2	0	2	0	2	1	2	1	2	2	1	2	2
(5) Ensured unbiased assessments through blinded evaluations.	0	0	0	0	0	0	0	0	0	1	0	0	0	2	0	1	2	0	2	1
(6) Ensured the follow-up duration aligns with the study's aims.	2	2	2	2	2	2	2	2	1	2	1	2	2	2	2	1	2	1	2	2
(7) Maintained a follow-up loss of less than 5% or included all patients.	0	1	2	2	1	1	0	1	1	2	1	1	2	1	2	1	1	1	1	1
(8) Calculated study size considering statistical relevance.	0	0	0	0	0	0	0	0	0	0	0	0	2	0	0	2	1	0	2	0
**For comparative studies also the next points:**																				
(9) Ensured an adequate control group in comparative studies.	1						2			2							0		0	
(10) Managed control and study groups simultaneously.	1						1			1							0		2	
(11) Ensured baseline equivalence between groups.	0						1			1							0		2	
(12) Utilized appropriate statistical analysis methods for reliability.	2						2			2							2		2	
**TOTAL MINORS score**	12	9	8	9	9	11	14	9	7	19	4	9	13	11	10	11	14	6	20	10
**Maximum possible score**	24	16	16	16	16	16	24	16	16	24	16	16	16	16	16	16	24	16	24	16

MINORS: Methodological index for non-randomized studies.
The items are scored 0 (not reported), 1 (reported but inadequate) or 2 (reported and adequate). The global ideal score being 16 for non-comparative studies and 24 for comparative studies.

Reference list of included articles

1. Aboul-Nour H, Alshaer Q, Khalid FC, et al. Anticoagulants versus Antiplatelet Treatment in the Medical Management of Carotid Floating Thrombus. *J Stroke Cerebrovasc Dis*. 2024;33(7):107760. doi:10.1016/j.jstrokecerebrovasdis.2024.107760

2. Bhogal P, AlMatter M, Aguilar Pérez M, Bäzner H, Henkes H, Hellstern V. Carotid Stenting as Definitive Treatment for Free Floating Thrombus—Review of 7 Cases. *Clin Neuroradiol*. 2021;31(2):449-455. doi:10.1007/s00062-020-00898-y

3. Chua HC, Lim T, Teo BC, Phua Z, Eng J. Free-floating thrombus of the carotid artery detected on carotid ultrasound in patients with cerebral infarcts: a 10-year study. *Ann Acad Med Singap*. 2012;41(9):420-424.

4. Combe J, Poinsard P, Besancenot J, et al. Free-floating Thrombus of the Extracranial Internal Carotid Artery. *Ann Vasc Surg*. 1990;4(6):558-562. doi:10.1016/S0890-5096(06)60839-X

5. M. M. M Cordier. Floating arterial thrombus in acute stroke treated with intravenous thrombolysis: Seven cases with mixed outcome . *Cerebrovascular Diseases*. 2012;33(Suppl. 2):1-2. doi:10.1159/000339538

6. Dowlatshahi D, Lum C, Menon BK, et al. Aetiology of extracranial carotid free-floating thrombus in a prospective multicentre cohort. *Stroke Vasc Neurol*. 2023;8(3):194-196. doi:10.1136/svn-2022-001639

7. El Harake S, Doche E, Bertolino J, et al. Symptomatic Carotid Free-Floating Thrombus: About Management of 50 Cases in a Referral Neurovascular Center. *J Clin Med*. 2023;12(23):7238. doi:10.3390/jcm12237238

8. Ferrero E, Ferri M, Viazzo A, et al. Free-floating thrombus in the internal carotid artery: Diagnosis and treatment of 16 cases in a single center. *Ann Vasc Surg*. 2011;25(6):805-812. doi:10.1016/j.avsg.2011.02.030

9. Jaberi A, Lum C, Stefanski P, et al. Computed tomography angiography intraluminal filling defect is predictive of internal carotid artery free-floating thrombus. *Neuroradiology*. 2014;56(1):15-23. doi:10.1007/s00234-013-1298-7

10. Lane TRA, Shalhoub J, Perera R, et al. Diagnosis and surgical management of free-floating thrombus within the carotid artery. *Vasc Endovascular Surg*. 2010;44(7):586-593. doi:10.1177/1538574410375312

11. Müller MD, Raptis N, Mordasini P, et al. Natural history of carotid artery free-floating thrombus—A single center, consecutive cohort analysis. *Front Neurol*. 2022;13. doi:10.3389/fneur.2022.993559

12. Naeem Khan MN, Ahmed A, Zafar I, Akhtar S, Aurangzeb MH, Khan A. The Diagnostic Accuracy of Carotid Doppler in Detecting Anechoic Thrombus Against CT Angiography as the Gold Standard. *Cureus*. 2022;14(7):e26951. doi:10.7759/cureus.26951

13. Onalan A, Gurkas E, Kursad Akpinar C, et al. Safety and effectiveness of anticoagulation in the management of acute stroke and transient ischemic attack due to intracranial and extracranial non-occlusive thrombus. *J Clin Neurosci*. 2024;124:47-53. doi:10.1016/j.jocn.2024.04.012

14. Panda S, Tiwari S, Pamnani J, et al. Large Vessel Occlusions By Free Floating Thrombi in Strokes During the COVID-19 pandemic- A Single Center Observational Study. *Neurol India*. 2022;70(2):623-632. doi:10.4103/0028-3886.344655

15. Pensato U, Forlivesi S, Gentile M, et al. Carotid free-floating thrombus in COVID-19: a cerebrovascular disorder of cytokine storm-related immunothrombosis. *Neurol Sci*. 2023;44(6):1855-1860. doi:10.1007/s10072-023-06682-3

16. Thornhill RE, Lum C, Jaberi A, et al. Can Shape Analysis Differentiate Free-floating Internal Carotid Artery Thrombus from Atherosclerotic Plaque in Patients Evaluated with CTA for Stroke or Transient Ischemic Attack? *Acad Radiol*. 2014;21(3):345-354. doi:10.1016/j.acra.2013.11.011

17. Tolaymat B, Irizarry K, Reif M, et al. Considerations beyond Stenosis for Carotid Endarterectomy in Treating Free-Floating Thrombus of the Carotid Artery. *Ann Vasc Surg*. 2019;60:221-228. doi:10.1016/j.avsg.2019.02.024

18. Torres C, Lum C, Puac-Polanco P, et al. Differentiating Carotid Free-Floating Thrombus From Atheromatous Plaque Using Intraluminal Filling Defect Length on CTA. *Neurology*. 2021;97(8):e785-e793. doi:10.1212/WNL.0000000000012368

19. Gülcü A, Gezer NS, Men S, Öz D, Yaka E, Öztürk V. Management of free-floating thrombus within the arcus aorta and supra-aortic arteries. *Clin Neurol Neurosurg*. 2014;125:198-206. doi:10.1016/j.clineuro.2014.08.008

20. Vassileva E, Daskalov M, Stamenova P. Free-Floating Thrombus in Stroke Patients with Nonstenotic Internal Carotid Artery-An Ultrasonographic Study. *J Clin Ultrasound*. 2015;43(1):34-38. doi:10.1002/jcu.22172
